# Endoplasmic reticulum stress in autoimmune skin diseases: immune activation, cutaneous cell damage, and therapeutic advances

**DOI:** 10.3389/fimmu.2026.1980040

**Published:** 2026-10-05

**Authors:** Jiaxi Qiao, Jia Chen, Jinbo Chen, Liuqing Chen

**Affiliations:** 1 Department of Dermatology, Traditional Chinese and Western Medicine Hospital, Hubei University of Chinese Medicine, Wuhan, Hubei, China; 2 Hubei Province & Key Laboratory of Skin Infection and Immunity, Wuhan, China

**Keywords:** autoimmune skin diseases, cutaneous immunology, endoplasmic reticulum stress, ERS–immune tolerance breaking, unfolded protein response

## Abstract

Endoplasmic reticulum stress (ERS) arises when the protein-folding load, or perturbations in calcium and redox homeostasis, outpace the adaptive capacity of the endoplasmic reticulum (ER). The unfolded protein response (UPR) initially reduces secretory load and restores proteostasis. Persistent stress can redirect the same pathways toward inflammatory signaling, altered antigen processing, and potentially immunogenic cell death. We term this context-dependent transition ERS–immune tolerance breaking, and propose it as a unifying framework for understanding how unresolved ERS may contribute to loss of cutaneous immune homeostasis. It is especially relevant in skin, where keratinocytes and melanocytes encounter ultraviolet radiation and oxidative injury while infiltrating immune cells face large biosynthetic demands. Evidence from vitiligo, pemphigus vulgaris, psoriasis, lupus erythematosus, and systemic sclerosis links dysregulated IRE1α, PERK, and ATF6 signaling to target-cell damage and immune amplification. In this review, we evaluate this evidence with particular attention to disease-specific mechanisms and the strength of causal inference supporting a pathogenic role for ERS. The case for a pathogenic role is strongest when ERS has been manipulated in defined cell types or experimental models; associations based only on UPR markers in lesions remain harder to interpret. Therapeutic studies also caution against treating the UPR as a uniformly harmful pathway. Intervention will probably need to preserve adaptive proteostasis while limiting the pathogenic output that predominates in a particular cell population and disease phase.

## 1 Introduction

As the primary site of secretory and membrane protein folding, the ER also governs calcium homeostasis, redox balance, lipid biosynthesis, and antigen processing — functions that become particularly consequential when cutaneous cells encounter autoimmune-mediated stress. Oxidative stress, ultraviolet irradiation, inflammatory cytokines, and autoantibody binding can disturb these functions and cause unfolded proteins to accumulate. Three transmembrane sensors then initiate the UPR: IRE1α (signaling through XBP1 splicing and RIDD), PERK (acting via eIF2α–ATF4–CHOP), and ATF6. **Figure 1** summarizes these branches and their adaptive and injury-associated outputs. Their early outputs lower translational demand and expand the capacity for folding and degradation, but the outcome depends on how long the signal persists: sustained signaling can engage NF-κB/JNK and the NLRP3 inflammasome, expose damage-associated molecular patterns (DAMPs), and activate cell-death programs (1, 2). In this review, ERS–Immune Tolerance Breaking describes this transition from an initially adaptive UPR to a response that supports immune recognition and loss of tolerance. We use the term as an analytical description, not as an established disease category.

**Figure 1:**
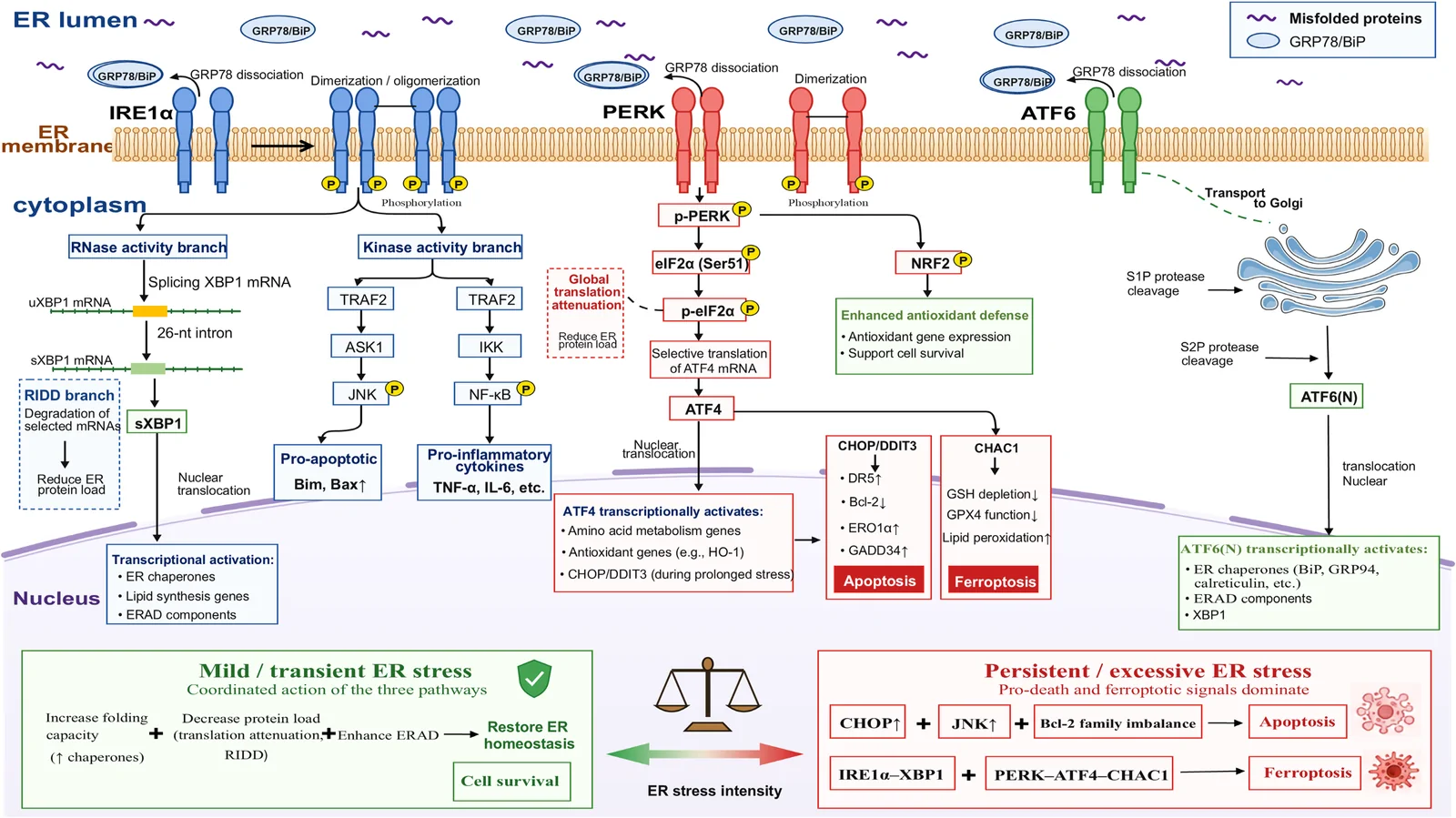
Canonical unfolded protein response signaling and cell-fate decisions during endoplasmic reticulum stress. Accumulation of misfolded proteins activates the three major UPR sensors, IRE1α, PERK, and ATF6, following dissociation of GRP78/BiP. IRE1α mediates XBP1 splicing, RIDD, and inflammatory signaling; PERK–eIF2α–ATF4 signaling reduces protein synthesis and promotes antioxidant adaptation, whereas sustained activation induces CHOP- and CHAC1-associated apoptosis and ferroptosis; ATF6 enhances ER chaperone expression, ERAD, and proteostasis. Mild or transient ERS favors adaptive repair and cell survival, whereas persistent or excessive stress shifts the UPR toward inflammatory, pro-apoptotic, and ferroptotic outputs. ER, endoplasmic reticulum; ERS, endoplasmic reticulum stress; UPR, unfolded protein response; GRP78/BiP, glucose-regulated protein 78/binding immunoglobulin protein; IRE1α, inositol-requiring enzyme 1 alpha; XBP1, X-box binding protein 1; RIDD, regulated IRE1-dependent decay; PERK, protein kinase R-like ER kinase; eIF2α, eukaryotic translation initiation factor 2 alpha; ATF4, activating transcription factor 4; ATF6, activating transcription factor 6; CHOP/DDIT3, C/EBP homologous protein/DNA damage-inducible transcript 3; CHAC1, ChaC glutathione-specific gamma-glutamylcyclotransferase 1; NRF2, nuclear factor erythroid 2-related factor 2; ERAD, ER-associated degradation; JNK, c-Jun N-terminal kinase; NF-κB, nuclear factor kappa B; TRAF2, TNF receptor-associated factor 2; ASK1, apoptosis signal-regulating kinase 1; IKK, IκB kinase; GSH, glutathione; GPX4, glutathione peroxidase 4; S1P, site-1 protease; S2P, site-2 protease.

Skin cells encounter different sources of ERS. Ultraviolet radiation, oxidants, and cytokines challenge keratinocytes, whose UPR influences barrier differentiation and immune-sentinel activity (3–5). Melanocytes combine a high melanin-synthesis load with substantial oxidative pressure and are correspondingly vulnerable (6–8). Matrix-producing fibroblasts face a different problem: protein overload in a fibrotic microenvironment (2, 9). Activated dendritic cells, lymphocytes, macrophages, and neutrophils must rapidly expand protein synthesis during differentiation or effector responses (10–14). Disease may therefore arise from the coincidence of stress responses in immune cells and injury in the cutaneous target cell; a uniform UPR defect shared by all compartments seems unlikely. **Figure 2** provides an overview of these immune-cell functions; the pathways shown differ in the strength of evidence linking them to skin autoimmunity.

**Figure 2:**
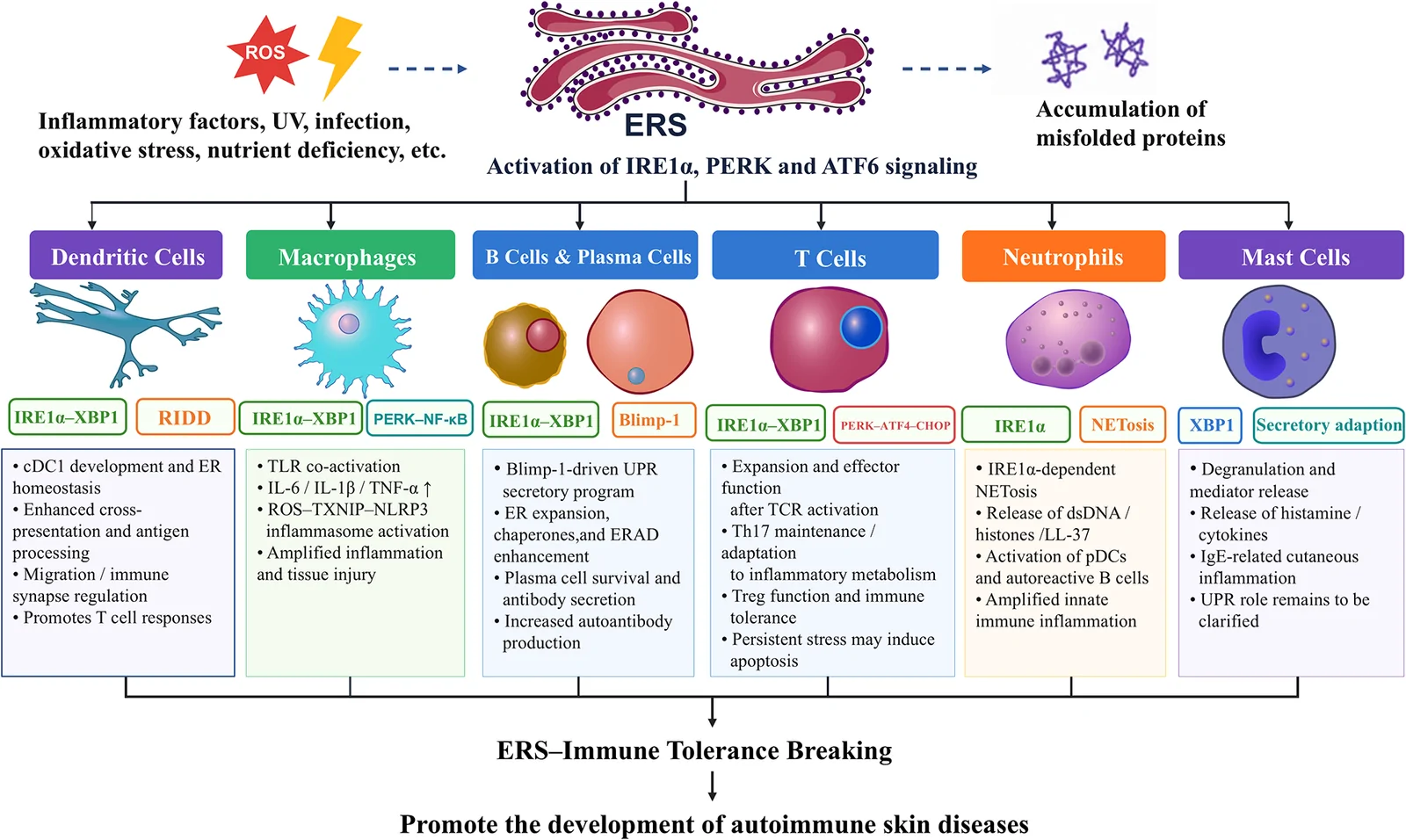
Endoplasmic reticulum stress reshapes immune-cell functions and contributes to immune tolerance breakdown in autoimmune skin diseases. Inflammatory mediators, ultraviolet radiation, infection, oxidative stress, nutrient deprivation, and other stressors induce endoplasmic reticulum stress (ERS) and activate the three major unfolded protein response (UPR) branches, including IRE1α, PERK, and ATF6 signaling. ERS exerts cell type-specific effects on dendritic cells, macrophages, B cells and plasma cells, T cells, neutrophils, and mast cells by regulating antigen presentation, cytokine production, antibody secretion, immune-cell differentiation and survival, NETosis, and secretory adaptation. Dysregulated or persistent UPR signaling may therefore disturb cutaneous immune homeostasis, facilitate loss of immune tolerance, and promote the development of autoimmune skin diseases. ERS, endoplasmic reticulum stress; UPR, unfolded protein response; ROS, reactive oxygen species; UV, ultraviolet radiation; IRE1α, inositol-requiring enzyme 1 alpha; XBP1, X-box binding protein 1; RIDD, regulated IRE1-dependent decay; PERK, protein kinase R-like endoplasmic reticulum kinase; ATF4, activating transcription factor 4; CHOP, C/EBP homologous protein; NF-κB, nuclear factor kappa B; Blimp-1, B lymphocyte-induced maturation protein 1; ERAD, endoplasmic reticulum-associated degradation; TLR, Toll-like receptor; IL, interleukin; TNF-α, tumor necrosis factor alpha; TXNIP, thioredoxin-interacting protein; NLRP3, NOD-like receptor family pyrin domain-containing 3; TCR, T-cell receptor; Th17, T helper 17 cell; Treg, regulatory T cell; NETosis, neutrophil extracellular trap formation; dsDNA, double-stranded DNA; LL-37, cathelicidin antimicrobial peptide LL-37; pDC, plasmacytoid dendritic cell; cDC1, conventional type 1 dendritic cell; IgE, immunoglobulin E.

The discussion begins with the UPR biology needed to interpret disease studies. We then compare the evidence in vitiligo, pemphigus vulgaris, psoriasis, cutaneous and systemic lupus erythematosus, and systemic sclerosis, distinguishing mechanistic experiments from descriptive changes in UPR markers. The therapeutic section uses the same distinction to assess chemical chaperones, branch-directed compounds, and downstream targets. Rather than asking whether ERS should be broadly activated or suppressed, we focus on a more precise question: which UPR outputs are pathogenic in specific cell types and disease stages?

## 2 ERS triggers activation of the UPR

### 2.1 Major triggers of ERS and ERS–immune tolerance breaking

The triggers of ERS are compartment-specific, reflecting the divergent biosynthetic demands and environmental exposures of cutaneous versus immune cells. UVB produces reactive oxygen species (ROS) and damages proteins in the keratinocyte ER (3, 4). In vitiligo melanocytes, H_2_O_2_ accumulation is a prominent trigger (7, 8). Cytokines such as IFN-γ and TNF-α, as well as anti-Dsg3 IgG, can translate extracellular inflammation or autoantibody binding into a keratinocyte stress response (15). Depletion of ER calcium stores through altered SERCA2 activity provides an endogenous trigger in keratinocytes (5). Plasma cells and other highly secretory cells instead face protein-folding pressure created by immunoglobulin production or high MHC-I expression (16, 17). The triggers above are compartment-specific, and consequently the significance of ERS differs from one skin disease to another.

Persistent ERS can couple proteostatic failure to inflammatory signaling and tissue injury (18–20). IRE1α-associated JNK activation and ERS-associated mitochondrial damage can promote inflammatory or cell-death outputs (21, 22). In skin models, chemokine release and surface calreticulin link stressed target cells to immune recruitment and antigen recognition (6, 23). Apoptosis and ferroptosis add autoantigens and damage signals to this response (24). ERS–Immune Tolerance Breaking refers to this functional boundary rather than to a single biochemical threshold. Operationally, we consider ERS to have entered a tolerance-breaking state only when persistent or maladaptive UPR activation is accompanied by both (i) a tissue-cell output that can increase immunogenicity—such as sustained inflammatory chemokine/cytokine release, DAMP or neoantigen exposure, altered antigen processing, or apoptosis/ferroptosis—and (ii) a demonstrable immune consequence, such as antigen-presenting-cell activation or cross-presentation, recruitment or expansion of autoreactive lymphocytes, or immune-mediated tissue injury that is reduced by genetic or branch-selective rescue. Sustained RIDD, persistent PERK–ATF4–CHOP signaling, or failure to restore ER homeostasis can support this assignment, but no single marker is sufficient. An isolated increase in BiP, XBP1s, phosphorylated eIF2α, or CHOP therefore indicates pathway engagement, not tolerance breaking. Stress duration, intensity, cell identity, and the linkage to immune recognition define the transition.

### 2.2 IRE1α–XBP1/RIDD signaling pathway

IRE1α — the most evolutionarily conserved ERS sensor — mediates both adaptive and proinflammatory outputs through its bifunctional kinase/ribonuclease domain. Dissociation of GRP78/BiP permits IRE1α oligomerization and activates its cytosolic ribonuclease. The best-characterized output is unconventional splicing of XBP1 mRNA. The resulting XBP1s protein increases chaperone expression, ER-associated degradation (ERAD), lipid synthesis, and ER expansion (1, 10). IRE1α also cleaves selected coding mRNAs and miRNAs through regulated IRE1-dependent decay (RIDD), including miR-150 (25). XBP1 splicing and RIDD are distinct readouts: the balance between them can separate adaptive ER expansion from loss of regulatory RNAs and tissue-specific pathology.

### 2.3 PERK–eIF2α–ATF4–CHOP signaling pathway

PERK reduces the influx of newly synthesized proteins into a stressed ER. After GRP78/BiP dissociation and autophosphorylation, PERK phosphorylates eIF2α at Ser51, suppressing most cap-dependent translation while allowing uORF-dependent translation of ATF4 (26, 27). During a transient response, ATF4 supports amino-acid metabolism and NRF2-dependent antioxidant defense. Prolonged activation changes the program. ATF4 induces CHOP/DDIT3, GADD34 restores translation before folding capacity has recovered, and CHAC1 lowers glutathione. Together, these events increase ROS, lipid peroxidation, and susceptibility to apoptosis or ferroptosis (28–32). PERK activity can thus be protective or harmful; phosphorylation alone does not establish which state is present.

### 2.4 ATF6 signaling pathway

ATF6 chiefly expands the ER’s folding and degradation capacity. After stress-dependent activation, it induces chaperones and ERAD genes and cooperates with XBP1 to support ER expansion and secretion (20, 33). In keratinocytes, ATF6-associated signaling has been linked to differentiation, barrier maintenance, and repair (5). Evidence that isolates ATF6 from the other UPR branches in autoimmune skin disease is still limited, so changes in ATF6 markers should be interpreted cautiously.

### 2.5 Crosstalk among UPR signaling pathways

The three UPR branches engage in extensive crosstalk — at the level of transcription, protein interaction, and metabolic output — rather than functioning as independent linear cascades (**Figure 1**). IRE1α can recruit TRAF2 and activate IKK, whereas PERK-mediated translational arrest reduces IκBα resynthesis; both routes can increase NF-κB activity (10, 34). ER-to-mitochondria calcium transfer, ROS, and TXNIP release connect ERS to the NLRP3 inflammasome and IL-1β/IL-18 production (22, 35). Autophagy counterbalances this inflammatory tone: PERK–ATF4 induces ATG genes and the CHOP–DR5 axis, while ER-phagy and aggregate clearance reduce the folding burden (14, 36, 37). Irreversible injury may follow when this feedback fails, independent of which sensor is activated.

How strongly, and for how long, these coupled pathways stay active determines the outcome. A brief response can preserve differentiation and repair, whereas severe or persistent stress increases chemokine release, DAMP and neoantigen exposure, and susceptibility to apoptosis or ferroptosis (5, 6, 10, 18, 23, 29, 38). This is why the same molecular marker may signal recovery in one disease phase and pathogenic amplification in another.

## 3 ERS–immune tolerance breaking in immune cells

Immune cells use the UPR for different tasks. In dendritic cells it affects antigen processing and cross-presentation; in plasma cells it supports sustained antibody secretion; in T cells it shapes metabolic adaptation and survival. Myeloid cells can couple IRE1α or PERK signaling to inflammasome activation and NETosis (10, 12–14, 38–40). Within the ERS–Immune Tolerance Breaking framework, these outputs cannot be collapsed into a single immune-cell stress state. Their direction depends on lineage, activation history, and which UPR branch dominates (**Figure 2**).

### 3.1 IRE1α–XBP1 in dendritic-cell antigen presentation

Antigen processing and cross-presentation place a persistent folding burden on dendritic cells (DCs) (12). The IRE1α–XBP1 axis is especially important in CD8α^+^ mouse and CD141^+^ human cDC1 cells. XBP1 deficiency disrupts ER homeostasis and cross-presentation, while continued IRE1α activity increases RIDD and alters antigen processing, migration, and immune-synapse molecules (12). Basal XBP1 splicing thus supports cDC1 function, whereas unrestrained RIDD can impair it. Whether this DC-intrinsic imbalance is sufficient to initiate cutaneous autoimmune disease, or instead amplifies responses triggered by target-cell stress, remains unresolved. In vitiligo, the better-supported mechanism is ER-stressed melanocytes promoting DC maturation and cross-presentation (6); evidence for a primary UPR defect intrinsic to DCs remains limited.

### 3.2 UPR control of antibody secretion in B cells and plasma cells

Plasma-cell differentiation is the clearest physiological example of a lineage-specific UPR (38, 39). Immunoglobulin secretion demands ER expansion, chaperone production, and ERAD, all supported by IRE1α–XBP1 (41, 42). ATF6 and PERK appear less prominent in this setting. The program is built into Blimp-1 (PRDM1)-driven terminal differentiation: Blimp-1 increases IRE1α/XBP1 expression and XBP1 splicing while limiting PERK–ATF4–CHOP-associated apoptosis (43, 44), thereby permitting antibody secretion to continue for the lifetime of the plasma cell. In autoimmune settings, however, evidence that UPR hyperactivation drives pathogenic autoantibody production remains largely correlative. Elevated GRP78 and XBP1 in SLE plasma cells is consistent with a high secretory load (45), but it does not by itself show that the UPR selected or sustained autoreactive clones.

### 3.3 UPR responses in T-cell activation and differentiation

T-cell receptor triggering drives a rapid expansion of protein synthesis and metabolic flux that imposes a substantial folding burden on the ER (13). IRE1α–XBP1 helps activated cells expand their folding capacity and can support proliferation and effector function (46, 47). This response is physiological, and on its own does not indicate pathogenic ERS. Disease relevance depends on whether the pathway changes lineage balance, tissue persistence, or tolerance.

Inflammatory hypoxia and nutrient restriction can increase XBP1s activity in Th17 cells and support their persistence (48). This connection is relevant to psoriasis and SLE, although direct evidence from skin-resident T cells is limited. PERK–ATF4 has a different role: moderate activity supports antioxidant defense, whereas persistent signaling induces CHOP and sensitizes cells to apoptosis (49). Tregs also require proteostasis. XBP1 contributes to their function, and excessive or inadequate UPR signaling may weaken suppression of Th17 and CD8^+^ responses (50–52). The available studies support context-dependent effects on T-cell fitness; they do not yet define a single UPR signature shared across autoimmune dermatoses.

### 3.4 UPR signaling in macrophages and neutrophils

Macrophages link protein-folding stress to innate inflammatory signaling. TLR stimulation uses IRE1α–XBP1 to increase cytokines such as IL-6, IL-1β, and TNF-α (10). Under persistent stress, PERK–NF-κB and IRE1α–TRAF2–JNK signaling can reinforce this response, while ROS/TXNIP activates NLRP3 and promotes IL-1β and IL-18 maturation (22). These interactions can sustain inflammation, but most evidence comes from general immune models rather than disease-specific skin macrophages.

Neutrophils provide more direct autoimmune evidence. In lupus models, abnormal IRE1α RNase activity promotes mitochondrial ROS, caspase-2 activation, and NETosis (14). NETs expose histones, dsDNA, LL-37, and other autoantigens that can stimulate autoreactive B cells and pDC TLR9 signaling (14, 53). Given that keratinocytes in lupus lesions also activate NLRP3-dependent inflammatory pathways, this NET-driven amplification may converge with epidermal innate signaling; however, the relative contribution of each compartment in human disease remains unquantified. For IRE1α inhibition this point is consequential: a benefit demonstrated in neutrophils cannot be extrapolated to macrophages or other cell types.

### 3.5 Evidence gaps in mast cells

Direct evidence connecting mast-cell UPR signaling to autoimmune skin disease is sparse. A recent mechanistic study showed that inhibition of the IRE1α–XBP1 axis suppresses IgE-dependent mast-cell degranulation, supporting a direct role for this branch in secretory activation (54); however, that work addressed allergic activation rather than autoimmunity. The decisive experiments in autoimmune skin disease are therefore lineage- and disease-specific. First, lesional, perilesional, and nonlesional skin should be compared by spatial co-detection of mast-cell markers (KIT/tryptase), XBP1s or phosphorylated IRE1α, and degranulation products. Second, primary human skin mast cells should be exposed to disease-relevant IgG/immune complexes, cytokines, or lesional conditioned media, with IRE1α/XBP1 perturbation and rescue while degranulation and cytokine output are quantified. Third, mast-cell-specific Ern1 or Xbp1 deletion should be tested in suitable autoimmune skin models to determine whether disease severity changes independently of general inflammation. If UPR activation accompanies mast-cell degranulation but lineage-restricted perturbation does not alter disease, mast cells should be regarded as bystanders rather than drivers.

## 4 Disease-specific evidence for ERS in autoimmune skin diseases

ERS does not occupy the same position in every autoimmune skin disease. We distinguish an initiator of autoreactivity from a proximal trigger of tissue injury and an amplifier of established disease. Human lesion studies establish localization and association; perturbation in cultured human cells supports a cellular mechanism; animal interventions support a role within that model. None alone establishes the temporal origin of human autoimmunity. **Table 1** separates these evidence settings, and each disease section ends with an explicit assessment of initiation versus amplification.

**Table 1:** Representative studies of ERS in autoimmune skin diseases.

Disease	Target cell	Model	UPR pathway	Major findings	Immune/pathological outcome	References
Vitiligo	Melanocyte	Primary human melanocytes	IRE1α–XBP1	4-TBP/MBEH activates IRE1α–XBP1 and induces IL-6 and IL-8	Local proinflammatory signaling	(59)
		Patient lesions/serum + *in vitro* model	PERK–eIF2α	Oxidative stress induces CRT overexpression and membrane translocation	DC maturation and CD8^+^ T-cell activation	(6)
		Patient samples + cellular experiments	PERK–CHOP	Homocysteine aggravates ERS-associated apoptosis	Autoantigen release	(8)
		Patient samples + *in vitro* model	SIRT1–UPR	SIRT1 deficiency aggravates ERS-induced apoptosis	Melanocyte loss	(58)
	Keratinocyte	Patient samples + cellular experiments	PERK + IRE1α	H_2_O_2_ induces CXCL16	Recruitment of CXCR6^+^ CD8^+^ T cells	(23)
Pemphigus vulgaris	Keratinocyte	Patient skin + *in vitro* model	IRE1α + PERK	The ER forms stable contacts with desmosomes/keratin; PV IgG induces Dsg3 rearrangement and activates the UPR	Desmosomal disruption and acantholysis	(15, 62)
		Patient serum + *in vitro* model	PERK–eIF2α	IgG-independent PERK activation	Altered cell cycle, viability, and adhesion	(61)
		*In vitro* model	PERK–CHOP	Upregulation of GRP94/BiP/CHOP	Impaired colony formation	(60)
Psoriasis	Keratinocyte	*In vitro* + animal models + patient tissue	IRE1α–XBP1s	Adaptive UPR is reduced in lesions; TM/BFA promotes differentiation and improves phenotype in the IMQ model	Correction of differentiation defects and suppression of abnormal proliferation	(70)
		Differentiating keratinocytes *in vitro*	Adaptive UPR	UPR activation accompanies keratinocyte differentiation	ERS inducers improve the psoriasis phenotype	(68)
		*In vitro* + animal models	GRP78–CCL20	Reduced GRP78 increases CCL20; GRP78 suppresses γδ T-cell migration	Regulation of CCR6^+^ IL-17A^+^ γδ T-cell recruitment	(74)
	Macrophage	*In vitro* + animal models	IRE1α–XBP1	Reduced SIRT3 enhances XBP1s acetylation and IL-23	Amplification of the IL-23/Th17 inflammatory axis	(73)
	DC	*In vitro* model	IRE1α–XBP1	ERS enhances stimulus-dependent IL-23 production by DCs	Th17 polarization	(72)
CLE/SLE	Keratinocyte	*In vitro* + animal models + patient scRNA-seq	UPR–SREBF–FPP–TRPV3	FPP activates TRPV3–Ca²^+^ signaling and induces IL-6 and CCL20	Enhanced migratory DC, Tfh, and germinal-center responses	(77)
		*In vitro* model + patient lesions	UVB-associated UPR and IFN signaling	UVB induces keratinocyte ERS, while the lupus-skin IFN milieu enhances UVB-induced apoptosis	Increased cell death and nucleic-acid/DAMP exposure	(75, 83)
	pDC	*In vitro* model	IRE1α–XBP1	XBP1s induces serine synthesis and reduces pyruvate/ATP	Restriction of ATP-dependent IFN-I production	(80)
	Neutrophil	*In vitro* + animal models	IRE1α	IRE1α promotes mitochondrial ROS and caspase-2-dependent NETosis	Increased NET formation and nuclear-antigen exposure	(14)
SSc	Fibroblast	*In vitro* + animal models + patient tissue	IRE1α–RIDD	RIDD degrades miR-150 and relieves repression of c-Myb/α-SMA	Myofibroblast activation and skin fibrosis	(25)
	pDC and fibroblast	*In vitro* model	PERK	ERS-stressed pDCs require direct contact to induce fibronectin/α-SMA in fibroblasts	Supports an immune-cell–fibroblast contact axis	(82)

### 4.1 Vitiligo

In vitiligo, oxidative stress links ER dysfunction to anti-melanocyte immunity. H_2_O_2_ activates PERK–eIF2α and IRE1α–XBP1 signaling in keratinocytes, increases CXCL16, and recruits CXCR6^+^ CD8^+^ T cells (23). Melanocytes respond differently: PERK-dependent translocation of calreticulin (CRT) to the surface promotes DC maturation and cross-presentation, enhancing autoreactive CD8^+^ T-cell activation (6, 55). Surface CRT also correlates with disease severity, although that association does not establish when ERS enters the disease course.

Several factors alter the threshold at which melanocyte stress becomes cytotoxic. Impaired NRF2 signaling weakens antioxidant buffering (56, 57). Homocysteine accumulation and low SIRT1 expression intensify PERK–eIF2α–CHOP signaling, apoptosis, and autoantigen exposure (8, 58). The vitiligo-inducing phenols 4-tert-butylphenol (4-TBP) and monobenzyl ether of hydroquinone (MBEH) instead activate IRE1α–XBP1 and induce IL-6 and IL-8 (59). Chemokine-mediated T-cell recruitment, CRT-dependent danger signaling, and melanocyte apoptosis are therefore likely to contribute at different phases and with different evidentiary strength. We place ERS closest to an initiating or bridging role in vitiligo: oxidative stress can precede overt immune infiltration and generate CXCL16- and CRT-dependent signals that recruit or activate autoreactive CD8^+^ T cells. Once the T-cell response is established, the same pathways are likely to amplify ongoing melanocyte injury. Longitudinal human data are still needed to prove this temporal sequence.

### 4.2 Pemphigus vulgaris

ERS-related transcripts are increased in pemphigus vulgaris (PV) skin (15). Anti-Dsg3 IgG raises GRP94, BiP, calnexin, and CHOP in keratinocytes and activates IRE1α and PERK; CHOP knockdown partially restores keratinocyte function (60). IgG-depleted PV serum can still induce PERK phosphorylation, indicating that non-IgG factors also contribute (61). Morphological studies add a structural explanation. ER tubules contact desmosomes and keratin filaments, and PV IgG increases contacts between the ER and internalized Dsg3 (15, 62). We interpret this desmosome–ER interface as a candidate stress-sensing structure, but its composition and causal role remain to be defined.

Among the proposed downstream mechanisms, p38 MAPK activation has the most direct connection to acantholysis. IFN-γ and anti-Dsg antibodies can act together through p38, with possible input from JAK–STAT and ERS signaling (63–65). CHOP-dependent apoptosis provides a second experimentally supported route to keratinocyte injury (60, 64, 65). By comparison, the suggestion that PERK-mediated translational arrest impairs renewal of Dsg1/3 or desmocollins is mechanistically plausible but has not been demonstrated directly. Oxidative markers and altered lipid metabolism in PV lesions support an additional feedback loop in which ROS aggravates ERS (66, 67), although its position relative to autoantibody binding is unresolved.

Collectively, the available data support a two-step model for PV pathogenesis: initial disruption of the desmosome–ER interface by autoantibody binding, followed by downstream UPR signaling that compromises keratinocyte adhesion and promotes apoptosis. Current evidence favors a role for ERS as a downstream amplifier rather than a primary initiator of autoimmunity in PV, because experimental studies predominantly place UPR activation after anti-Dsg antibody binding or exposure to other pathogenic serum factors (15, 60–62). ERS may therefore magnify acantholysis and keratinocyte injury without explaining the upstream loss of B-cell tolerance that generates pathogenic antibodies.

### 4.3 Psoriasis

Psoriasis reveals opposing UPR states across cell types. Adaptive signaling is reduced in keratinocytes, whereas inflammatory UPR outputs can be increased in immune cells. Lesional epidermis shows lower GRP78/BiP, CHOP, HRD1, IRE1α–XBP1s, PERK, and related signals (68–70). Physiological UPR activity normally accompanies epidermal differentiation: ATF6 participates in calcium signaling and lysosomal biogenesis, while IRE1α–XBP1s supports C/EBPβ, KLF4, ABCA12, and barrier formation (5, 68, 71). Reduced activity of these programs offers a plausible explanation for persistent proliferation and incomplete differentiation, although marker loss in established lesions does not prove that it was the initiating defect.

Experiments with ERS inducers provide unusually direct, if counterintuitive, support for this model. Tunicamycin and brefeldin A improve the phenotype of imiquimod-treated mice and restore XBP1-dependent ABCA12 and KLF4 expression (68). These findings indicate that boosting adaptive UPR signaling in keratinocytes can ameliorate psoriasiform inflammation, but this effect is compartment-specific and does not justify broad ERS activation as a therapeutic strategy. In DCs and macrophages, IRE1α–XBP1 can increase IL-23, while hypoxia and glucose restriction support Th17 adaptation (48, 72, 73). Prolonged GRP78 deficiency may also increase CCL20 and recruit γδ T cells (70, 74). Current evidence supports compartment-specific amplification, through deficient adaptive UPR in keratinocytes and inflammatory outputs in myeloid cells, rather than a single initiating ERS lesion in psoriasis. Deficient adaptive UPR in keratinocytes may create a permissive state for abnormal differentiation, whereas IRE1α–XBP1 activity in DCs and macrophages is more plausibly an amplifier of an established IL-23/Th17 circuit. Treatment design must separate keratinocyte differentiation from immune-cell inflammatory outputs and account for disease phase.

### 4.4 Cutaneous and systemic lupus erythematosus

Ultraviolet (UV) injury links keratinocyte stress to photosensitive inflammation in cutaneous and systemic lupus erythematosus. ROS generation and ER calcium loss activate PERK and IRE1α, promoting apoptosis and release of nucleic acids (3, 75, 76). Those nucleic acids can stimulate pDCs through cGAS–STING and TLR7/9, increasing type I interferon. The proposed UV–ERS–nucleic-acid pathway is biologically coherent, but human data mostly come from established lesions and do not yet resolve whether UPR activation precedes the interferon-rich environment or is reinforced by it.

ERS may also connect epidermal injury to humoral immunity through metabolism. UV-induced UPR–SREBP–mevalonate signaling increases farnesyl pyrophosphate (FPP) and activates TRPV3. Ca²^+^–NFAT/ERK signaling then induces IL-6 and CCL20, promoting migratory DC, T follicular helper, germinal-center, and plasma-cell responses (77). We regard this pathway as conceptually important because it offers a route from local keratinocyte stress to systemic autoantibody production— a conceptual extension beyond the familiar DAMP-release paradigm. Separate observations of elevated GRP78 and XBP1 in SLE plasma cells are consistent with high-rate antibody secretion but do not prove a causal role (45). Neutrophil IRE1α-driven NETosis provides an additional source of nuclear antigen and pDC–TLR9 activation (14, 53).

Together, these observations point to a sequential model rather than three parallel, equipotent pathways. UV-exposed keratinocytes supply nucleic acids and metabolic signals that activate pDCs and support B-cell help. NETosis can then enlarge the nuclear-antigen pool and reinforce immune-complex formation. ERS sits at the conversion step —the point where a physical exposure becomes a signal the immune system can recognize. The relative importance of keratinocyte UPR, NETosis, and plasma-cell adaptation is likely to vary between cutaneous lesions and systemic disease. In lupus, ERS is therefore best viewed as a proximal transducer of UV injury and an amplifier of interferon, NET, and humoral circuits, rather than a demonstrated initiator of systemic autoimmunity. It may contribute to lesion initiation after UV exposure in susceptible skin, but evidence that ERS precedes the systemic loss of tolerance is lacking.

### 4.5 Systemic sclerosis

In systemic sclerosis (SSc), the most direct evidence concerns IRE1α RNase activity in fibroblasts. TGF-β increases XBP1s and RIDD-mediated loss of miR-150, releasing c-Myb–α-SMA signaling and promoting myofibroblast differentiation (25). Genetic disruption of IRE1α or pharmacological inhibition with 4μ8C attenuates this phenotype (25). By comparison, the evidence for pDC involvement is less settled: TLR7/8 activation drives IFN-α and CXCL4 (78, 79), while IRE1α–XBP1s–PHGDH and NRF2 can restrain IFN-I production; excessive CXCL4 may weaken that brake (80, 81). PERK-dependent stress in pDCs has also been reported to induce fibronectin and α-SMA in fibroblasts through direct contact (82).

Fibroblast IRE1α–RIDD signaling currently has the clearest experimental support as an ERS-dependent fibrotic mechanism. Persistent pDC activation and pDC–fibroblast contact may reinforce it, but their contribution in human skin has not been quantified. This evidence supports a multicellular feedback model without implying that all three UPR branches are uniformly activated. In SSc, ERS is most convincingly positioned as an amplifier and maintenance mechanism: fibroblast IRE1α–RIDD can sustain fibrosis and pDC–fibroblast crosstalk can reinforce it, but there is no direct evidence that ERS initiates the upstream autoimmune state.

## 5 Therapeutic strategies: evidence and risks

### 5.1 Chemical chaperones and proteostasis enhancers

By reducing misfolded protein load non-specifically, chemical chaperones such as 4-phenylbutyrate (4-PBA) and tauroursodeoxycholic acid (TUDCA) attenuate ER stress without engaging a single UPR branch. In MRL/lpr mice, 4-PBA reduces anti-dsDNA antibodies, renal immune-complex deposition, and inflammatory cytokines (84), but this systemic model does not establish efficacy against cutaneous lupus or identify the responding skin cell. TUDCA protected against aggregation and stress-associated death in a hepatoma-cell system (85), which supplies mechanistic precedent rather than evidence of benefit in autoimmune skin disease. Because both compounds have additional actions, improvement should be linked to target-cell survival or immune function as well as UPR readouts. A plausible use in active vitiligo or PV would be protection of surviving target cells during ongoing injury, but that proposal remains untested clinically and should not be generalized to psoriatic keratinocytes with deficient adaptive signaling.

### 5.2 Branch-directed modulation of IRE1α and PERK

IRE1α-directed intervention is most compelling when a harmful RNase output is identified in a defined compartment. In SSc fibroblast models, inhibition can limit RIDD-associated miR-150 loss and myofibroblast differentiation (25); in lupus, IRE1α inhibitors reduce NET-associated outputs in human neutrophil experiments and mouse models (14). These results support investigation during active fibrogenesis or inflammatory amplification, respectively, but they do not establish treatment of human skin disease, reversal of fixed fibrosis, or prevention of autoreactivity. Complete IRE1α inhibition may also impair adaptive XBP1 signaling and B-cell tolerance (86).

Partial antagonists of the IRE1α RNase (PAIRs) demonstrate that XBP1 splicing and damaging RNase outputs can be pharmacologically separated in experimental systems (87). Kinase-inhibiting RNase attenuators, including KIRA7 and KIRA8, showed antifibrotic effects in a bleomycin lung model (88); these findings provide a rationale for further study but do not establish efficacy in SSc skin. For a skin-directed candidate, XBP1 splicing and RIDD substrates should be measured separately to establish the intended output profile. Sparing XBP1 could preserve epidermal adaptation but might also preserve XBP1-dependent IL-23 production in myeloid cells (72, 73), so output selectivity cannot substitute for cell selectivity.

PERK inhibition is a candidate for reducing CHOP-associated target-cell injury in experimental vitiligo or PV (8, 60, 89). However, phosphorylated eIF2α and ATF4 are outputs of the wider integrated stress response and do not uniquely identify PERK. GSK2606414 and GSK2656157 also inhibit RIPK1 (90). A convincing treatment effect therefore requires PERK-specific perturbation, rescue or an independent compound, and simultaneous evidence that adaptive translation control and tissue viability are preserved. In psoriasis, restoration of adaptive epidermal UPR activity may be more appropriate than inhibition (70); in SSc pDCs, suppressing an adaptive UPR brake could instead increase interferon output (80).

### 5.3 Downstream targets at the tissue–immune interface

Downstream targets may avoid some of the systemic consequences of inhibiting an entire UPR branch, but the evidence is uneven. Blocking CXCL16–CXCR6 could limit epidermal recruitment of CXCR6^+^ CD8^+^ T cells in vitiligo (23). TRPV3 antagonism is a proposed way to interrupt the FPP–TRPV3–IL-6/CCL20 pathway in SLE (77), although this strategy has not been tested clinically. Ruxolitinib can reduce IFN-γ–JAK–STAT signaling and indirectly attenuate PERK/eIF2α/ATF4/CHOP activation in pemphigus models (89). NRF2 activators, including tapinarof and dimethyl fumarate, strengthen antioxidant defense, but their clinical effects should not be attributed solely to ERS modulation (57, 91). What recommends these targets is their proximity to a defined disease mechanism, but this advantage remains theoretical until target-engagement data are generated in the relevant skin cell.

### 5.4 Biomarkers and patient stratification

XBP1 splicing, phosphorylated eIF2α, ATF4/CHOP, and GRP78/BiP are useful research readouts, but none is a validated clinical biomarker of pathogenic ERS. Translating these therapeutic targets into clinical practice will require validated biomarkers that can distinguish pathogenic from adaptive ERS in patient tissue. Their interpretation is complicated by cell mixing in biopsies and by the fact that the same marker can accompany adaptation or injury. Disease-associated measures may be more informative: CXCL16 tracks oxidative stress and activity in vitiligo (23), neutrophil IRE1α RNase activity correlates with SLEDAI in SLE (53), and serum miR-150 is inversely associated with skin fibrosis in SSc (25). Prospective studies must test whether these measures predict treatment response beyond established clinical markers. Spatial and single-cell assays can identify the relevant compartment, but as discovery tools they do not yet amount to an ‘ERS molecular classification’. Pre-analytical standardization is essential before defining cutoffs or comparing centers (Section 6.1).

### 5.5 Safety and biological trade-offs

UPR-directed interventions carry inherent risks in tissues with high secretory demand, a constraint that must be weighed against any potential benefit in autoimmune skin disease. Systemic inhibition may impair plasma-cell antibody secretion or pancreatic β-cell proteostasis (41, 92), and complete IRE1α blockade may disturb B-cell tolerance (86). Excessive enhancement carries different risks, including tumor-cell survival (93–95), IRE1α–XBP1-associated fibrosis (25), and IL-23 production by DCs or macrophages (72, 96, 97). These observations do not define a simple balance between too much and too little UPR activity. Instead, each of dose, duration, cell exposure, and branch-specific target engagement will have to be measured directly in preclinical and early clinical studies.

Topical or other skin-directed delivery is a realistic development strategy for epidermal targets because it could achieve local exposure while reducing systemic drug levels. Topical/transdermal 4-PBA improved re-epithelialization and reduced ERS markers in a human ex vivo skin-wound model (98), although this does not establish efficacy in autoimmune disease. Direct UPR modulators still require evidence of penetration into viable epidermis or dermis, intracellular target engagement, and a suitable skin-to-plasma exposure profile. Barrier disruption, treated surface area, occlusion, and repeated dosing can alter absorption; topical administration therefore cannot be assumed to eliminate systemic exposure.

## 6 Limitations and research priorities

Most causal evidence in this field comes from cell culture or genetically engineered mice; human studies are usually cross-sectional—and the weight of this limitation varies across diseases. In vitiligo and pemphigus, increased BiP or CHOP in an established lesion may reflect ongoing injury rather than its cause. In psoriasis, bulk-tissue measurements can obscure opposite responses in keratinocytes and infiltrating immune cells. Lupus and systemic-sclerosis biopsies are commonly obtained after interferon signaling or fibrosis is already established, making temporal order difficult to infer. Pre-analytical variables constitute an additional source of uncertainty: phosphorylation states and XBP1 splicing ratios can shift substantially during tissue collection, processing, and storage. Phosphorylation can change within minutes after excision because ischemia itself perturbs stress-kinase activity, and delayed or formalin fixation can alter phosphoepitope recovery (99, 100). XBP1 splicing is an RNA-based ratio whose interpretation depends on RNA integrity, rapid stabilization, and isoform-specific assay design; spliced and total/unspliced XBP1 should be measured directly rather than inferring IRE1 activity from total XBP1 expression (101). For human studies, biopsy-to-preservation time, temperature, fixation duration, storage history, and freeze-thaw cycles should be prospectively recorded and standardized. Ideally, each biopsy would be divided immediately for snap-frozen or RNA-stabilized analysis, phosphoprotein-preserving assays, and histology, with marker-specific stability validated before clinical interpretation. A single UPR marker cannot establish causality or distinguish an initiating event from a secondary adaptive response (1, 18).

Pharmacological evidence requires similar caution. 4-PBA and TUDCA are pleiotropic, and GSK2606414 inhibits RIPK1 in addition to PERK (90, 102, 103). A result obtained with one tool compound should not be assigned to a UPR branch unless it is reproduced with genetic perturbation, rescue, or a chemically distinct inhibitor. Current skin equivalents and organoids also fail to preserve the full interaction among immune cells, nerves, vasculature, fibroblasts, and extracellular matrix. Models that combine defined cell populations with longitudinal sampling would be more informative than another cross-sectional marker survey.

One specific gap is stress-dependent ERAP1/ERAP2 peptide processing and MHC-I loading, which deserves separate attention because it offers a mechanistic bridge between proteostasis and autoimmune recognition. ERAP1 and ERAP2 trim N-terminally extended peptides in the ER and thereby shape the MHC-I immunopeptidome. ERS could influence this axis even without directly changing ERAP transcription: PERK-mediated translational attenuation and ER-associated degradation alter the pool of peptide precursors, while changes in ER chaperones and peptide-loading machinery can modify MHC-I assembly (104–108). In psoriasis, ERAP1-dependent processing regulates the amount of a melanocyte autoantigen presented by HLA-C*06:02 and the resulting autoreactive T-cell response (106). Human skin single-cell studies also associate disease and genetic variation with cell-specific ERAP1/ERAP2 expression patterns (107). ER stress has also been shown in non-cutaneous systems to alter MHC-I peptide presentation (109). These are stronger connections to antigen recognition than a generic stress marker, but neither demonstrates that ERS controls ERAP activity in keratinocytes or melanocytes. A high-priority experiment would combine branch-selective UPR perturbation in HLA-typed primary keratinocytes or melanocytes with ERAP1/2 activity assays, quantitative immunopeptidomics, surface MHC-I measurements, and antigen-specific CD8^+^ T-cell readouts. ERAP1/2 knockout and rescue would distinguish a true peptide-trimming effect from a general reduction in protein synthesis or peptide supply.

## 7 Conclusion

Across autoimmune skin diseases, ERS occupies different positions in the pathogenic sequence. In vitiligo it may operate near disease initiation by converting oxidative injury into CXCL16/CRT-dependent immune recruitment, although longitudinal human proof is incomplete. In PV, ERS is more clearly downstream of autoantibody binding and amplifies keratinocyte injury. In psoriasis, deficient adaptive UPR may create a permissive keratinocyte state, whereas myeloid IRE1α–XBP1 amplifies IL-23/Th17 inflammation. In cutaneous and systemic lupus erythematosus, ERS mainly translates UV injury into interferon, metabolic, and NET-associated amplification; in SSc, fibroblast IRE1α–RIDD and pDC–fibroblast crosstalk sustain fibrosis rather than explain the initial loss of tolerance. This disease-by-disease positioning is more informative than treating ERS as a uniform upstream cause.

The central question is therefore not whether the UPR is activated, but which UPR output is pathogenic in which cell type and at which disease stage. ERS–Immune Tolerance Breaking is useful only when it is applied operationally to this transition: persistent stress must be linked to an immunogenic tissue-cell output and a demonstrable immune consequence, rather than inferred from a single UPR marker. Longitudinal skin sampling, lineage-restricted perturbations, and orthogonal pharmacology would clarify which UPR outputs precede tissue injury and which are secondary. Clinical translation will also require selective compounds and pharmacodynamic readouts in the affected compartment. Until such data are available, ‘restoring ER homeostasis’ remains a testable therapeutic hypothesis, and its translation into a single intervention applicable across diseases cannot be assumed.
